# Unique Mode of Lipogenic Activation in Rat Preputial Sebocytes

**DOI:** 10.1155/2011/163631

**Published:** 2011-07-26

**Authors:** Dianne Deplewski, Kenan Qin, Nancy Ciletti, Robert L. Rosenfield

**Affiliations:** Department of Pediatrics, Pritzker School of Medicine, The University of Chicago, 5841 S. Maryland Avenue (MC-5053), Chicago, IL 60637-1470, USA

## Abstract

Lipoprotein delivery of fatty acids and cholesterol is linked with peroxisome proliferator-activated receptor (PPAR) activation in adipocytes and macrophages. We postulated that similar interactions exist in sebaceous epithelial cells (sebocytes) in which PPAR activation induces differentiation. High-density lipoprotein (HDL) and very low-density lipoprotein (VLDL) markedly enhanced sebocyte differentiation above that found with PPAR agonists and were more potent than explicable by their lipid content. The PPAR**γ** antagonist GW5393 reduced sebocyte differentiation to all PPAR isoform agonists, HDL and VLDL, suggesting that the lipoprotein effect on differentiation occurs partially through activation of PPAR**γ**. Furthermore, we found that sebocytes expressed a unique pattern of lipogenic genes. Our results demonstrate that HDL and VLDL are the most potent inducers of sebocyte differentiation tested to date, and these actions are partially inhibited by PPAR antagonists. This suggests that substrates provided by lipoproteins are targeted to sebocytes and affect their own disposition via PPAR activation.

## 1. Introduction

Sebaceous epithelial cell (sebocyte) differentiation is defined by increasing accumulation of lipid droplets, the major component of sebum. Peroxisome proliferator-activated receptor (PPAR) isoforms, which were originally discovered because of their key roles in adipogenesis and lipid metabolism, have been shown to strongly stimulate sebocyte differentiation in vitro [1–4]. All PPAR isoforms have been identified in sebocytes [1, 2, 5]. PPAR*γ* seems essential for sebocyte differentiation, as sebocytes do not appear to develop from cell lineages devoid of PPAR*γ* [6], and PPAR*γ* agonists induce lipid-droplet forming colonies (LFCs) in the primary rat preputial cell culture model of sebocyte differentiation but not in cultured epidermal cells (keratinocytes) [1]. PPAR*δ* has been deduced to be important in sebocyte differentiation since linoleic acid and carbaprostacyclin (cPGI2), which are agonists of both PPAR*α* and PPAR*δ*, induce more LFCs in sebocytes than either PPAR*α* or PPAR*γ* agonists [1]. Linoleic acid is the most effective stimulator of sebocyte differentiation tested thus far [1, 2], but its physiological relevance is suspect since its action requires a relatively high concentration (10^−4^ M), and it inappropriately stimulates LFCs in epidermal cells [1], which raises the possibility that it mainly acts as a fatty acid substrate for lipogenesis.

Recently, PPAR action in macrophages and adipocytes has been shown to be intimately linked with lipoprotein delivery of fatty acids and cholesterol [7–14]. The relationship is complex, and the actions of PPAR isoforms may be interactive. For example, while macrophage uptake of low-density lipoprotein (LDL)-derived fatty acid metabolites is PPAR*δ*-inducible [13], it is PPAR*γ*-dependent [7–9], yet very low-density lipoprotein (VLDL)-induced triglyceride accumulation in macrophages is absolutely PPAR*δ*-dependent [14]. Lipoprotein is among the lipogenic regulators shown to be important for sebaceous differentiation in studies of genetically modified mice [15].

These considerations led us to test the hypothesis that lipoproteins interact with PPAR*γ* and PPAR*δ* to induce sebocyte differentiation in a unique pattern that is related to a distinctive pattern of gene expression. Our studies are compatible with the concept that lipoproteins act to target lipid substrates within skin to sebocytes, where they induce differentiation via PPAR-mediated lipogenic pathways.

## 2. Material and Methods

*Cell Preparations and Culture*. All animal experimentation was conducted in accordance with accepted standards of humane animal care. Single-cell suspensions of rat preputial sebocytes (or epidermal cells for control purposes) obtained from 60 d.o. male Sprague-Dawley rats were prepared by enzymatic digestion under sterile conditions, as previously reported [16]. Single-cell suspensions were plated on growth-arrested 3T3-J2 fibroblast feeder layers in Dulbecco's Modified Eagles Medium (DMEM) with 10% fetal calf serum supplemented with 10^−6^ M insulin, 10^−10^ M choleratoxin, 10^−6^ M cortisol, and antibiotics, as previously reported [1]. The cells were grown in primary culture in 95% air/5% CO_2_ at 37°C. A serum-free chemically defined cell culture medium (Cellgro Complete) was substituted for DMEM plus serum after the 3rd day of culture. Treatments were added in triplicate for 48 hours on day 7 (preconfluence). Treatments included the PPAR activators troglitazone (Tro; specific activator of PPAR*γ*), cPGI2 (activator of PPAR*δ*, *α*), and linoleic acid (activator of PPAR*δ*, *α*). In addition, we used the specific PPAR*δ* agonists GW610742 (GW742) [17], and L-165041 [18], the specific PPAR*γ* agonist GW347845 (GW845) [19], the specific PPAR*γ* competitive antagonist GW5393 [17] and the noncompetitive PPAR*γ* binding pocket antagonist GW9662 [20] that were kindly provided by GlaxoSmith-Kline and Merck, courtesy of Drs. Tim Willson and Joel Berger, respectively. Agonists and antagonists were prepared as a stock solution at 10 mM in DMSO and stored in aliquots at −20°C. The antagonists were added to incubation medium 2 hours before agonists. Human lipoproteins were purchased from EMD Biosciences. For histochemical determination of sebocyte differentiation, cells were grown in 35 mm 6-well plates. For RNA preparations, Trizol reagent was either added directly to single-cell suspensions or to cultured cells attached to the plate.

*Histological Analysis of Lipid Droplet Formation*. LFCs were quantified by light microscopy after Oil Red O staining, as previously described [21]. A LFC is defined as a colony containing over 5 cells positive for Oil Red O staining (cells equivalent to at least middifferentiation of sebocytes), in order to clearly distinguish specific cytoplasmic staining from the amorphous staining of deteriorating cells, as previously described [1]. 

*Triglyceride/Cholesterol Assays*. Triplicate wells from selected treatments were washed in Dulbecco's phosphate-buffered saline (PBS) three times and then trypsinized, collected, and resuspended in PBS. The cell suspensions were analyzed for triglyceride and cholesterol content. A UV method for the determination of triglycerides was used according to the manufacturer (Test-Combination Glycerol, Roche). In a series of enzymatic reactions, NADPH was determined by means of its light absorbance at 340 nm and was directly related to the amount of glycerol or triglycerides present in the cell suspension. The cholesterol content was determined by a colorimetric method (Test-Combination Cholesterol, Roche) utilizing cholesterol oxidase and reagents that generate a lutidine-dye, that is, stoichiometric to the amount of cholesterol in the sample and was measured at 405 nm. Standard curves were generated for each experiment to determine the accuracy of the assay. 

*Reverse Transcriptase Polymerase Chain Reaction*. RT-PCR was carried out as previously described [22] with minor modifications according to the manufacturer's protocol (from SuperScript First-Strand Synthesis System for RT-PCR, Gibco BRL). Briefly, single-stranded cDNA was synthesized from 2 *μ*g total RNA using reverse transcriptase and random primers in 20 *μ*L. RT-PCR for lipogenic gene expression was performed using specific primers (Table 1). Two *μ*l of single-stranded cDNA in a total 50 *μ*L PCR reaction mixture was denatured for 5 min at 95°C, then thirty-five cycles of PCR amplification were performed under the following conditions: 15 sec at 94°C, 15 sec at 50°C, and 2 min at 72°C, finally the samples were placed for 7 min at 72°C. A negative control was carried out for each pair of primers with the same procedure without reverse transcriptase. Amplified DNA was resolved on a 1.5% agarose gel containing 5 *μ*g/mL ethidium bromide and bands visualized under UV light.

*Statistical Analysis*. One-way ANOVA followed by Fisher's Protected Least Differences post hoc test was used to compare the various treatments. Statistical analyses were performed using the Statview program; a *P*-value (two-tailed) <0.05 was considered statistically significant.

## 3. Results

### 3.1. Specific PPAR*δ* and PPAR*γ* Agonists Induce Sebocyte Differentiation

**Figure 1:**
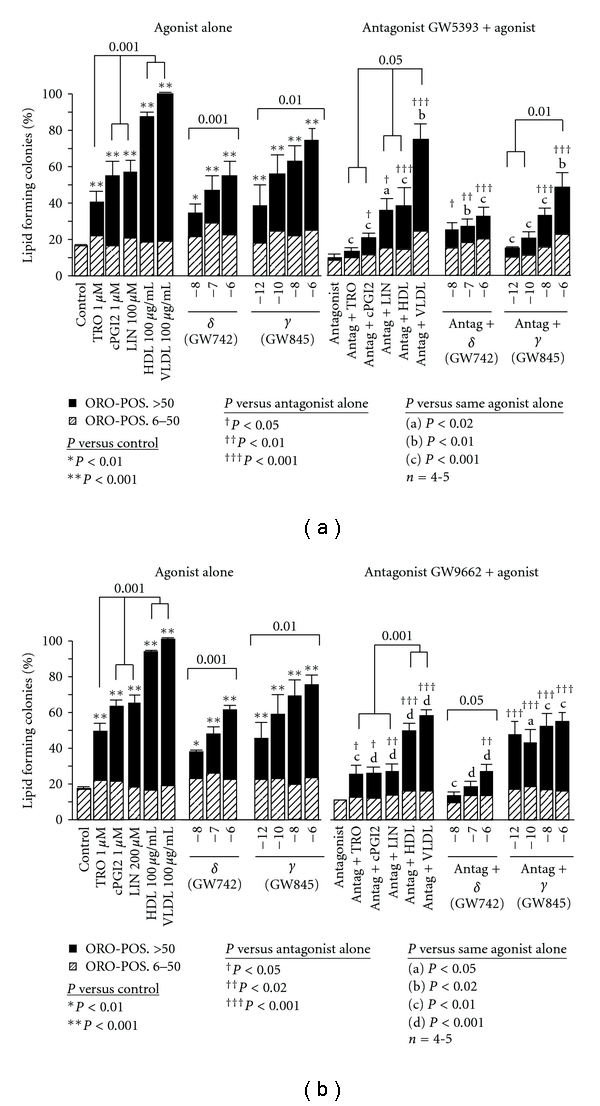
Comparison of sebocyte differentiation induced by PPAR agonists and lipoproteins before and after pretreatment with two PPAR antagonists. Maximally effective doses of the selective PPAR agonists troglitazone (TRO), carbaprostacyclin (cPGI2), and linoleic acid (LIN) were used as indicated. HDL and LDL were used at 100 *μ*g protein/mL. The specific PPAR*δ* (GW742) and PPAR*γ* (GW845) agonists were used at the doses indicated. The PPAR*γ* antagonist GW5393 (a) and the PPAR binding pocket antagonist GW9662 (b) were added to the cells at a dose of 1 *μ*g 2 hours prior to treatment with the PPAR agonists or lipoproteins on day 7 of primary culture. LFC determination was made on day 9 of culture after fixing and staining the cells with Oil Red O (ORO). Striped bars indicate colonies with 6–50 ORO-stained cells, and solid bars those colonies with >50 ORO-stained cells. Means +/− SEMs are presented.

Recently available specific PPAR*δ* agonists were used to distinguish the role of PPAR*δ* from that of PPAR*α* in sebocyte differentiation. We show that the specific PPAR*δ* agonist (GW742) exhibits a dose-response effect on sebocyte differentiation (*P* < 0.001; Figure 1) and is equipotent to the PPAR*δ*, *α* agonist cPGI2 at 1 *μ*M. A similar statistically significant dose-response effect on sebocyte differentiation was found with the specific PPAR*δ* agonist L-165041 (data not shown). These results support a specific role of PPAR*δ* in sebocyte lipogenesis. 

The specific PPAR*γ* agonist (GW845) also exhibited a dose-related effect on sebocyte differentiation (*P* < 0.01; Figure 1). Furthermore, it was the most potent of all PPAR isoform agonists tested. (*P* < 0.01; Figure 1). These results add further evidence of the importance of PPAR*γ* activation on sebocyte differentiation.

### 3.2. PPAR*γ* Antagonists Block PPAR*γ* and PPAR*δ* Action in Sebocytes

The specific PPAR*γ* competitive antagonist GW5393 at 1 *μ*M specifically inhibited the response to all PPAR*γ* agonists: the LFC responses to the lowest doses of the potent specific PPAR*γ* agonist (GW845) and the low potency troglitazone were completely blocked, and those to higher doses of GW845 were significantly inhibited (Figure 1(a)). As expected, LFC formation in response to the lowest dose of the specific PPAR*δ* agonist GW742 was not significantly inhibited by GW5393. However, the effect of higher doses of the PPAR*δ* agonist and of the nonspecific PPAR*δ*,*α* agonists cPGI2 and linoleic acid were partly inhibited by GW5393. We interpret this as indicating that low-level PPAR*δ* stimulation induces sebocyte lipogenesis and that higher level PPAR*δ* stimulation of lipogenesis involves signaling via PPAR*γ*. 

The PPAR*γ* binding pocket antagonist GW9662 completely blocked the effect of the specific PPAR*δ* agonist GW742 at the lower doses and did not inhibit the effect of the specific PPAR*γ* agonist GW845 at the lowest dose (Figure 1(b)). Although GW9662 behaves as a relatively specific PPAR*γ* antagonist in adipocytes [20], these studies indicate that GW9662 acts functionally as a specific inhibitor of PPAR*δ* in sebocytes. This suggests that the apparent cell-specific effects of GW9662 are dependent on cell-specific expression of coregulator molecules. GW9662 inhibited the responses to higher doses of both these agonists and to all other PPAR activators. These data support the concept of cooperation in the actions of PPAR*δ* and PPAR*γ* in sebocyte lipogenesis.

### 3.3. The Lipoproteins HDL and VLDL Are Potent Inducers of Sebocyte Differentiation

**Figure 2:**
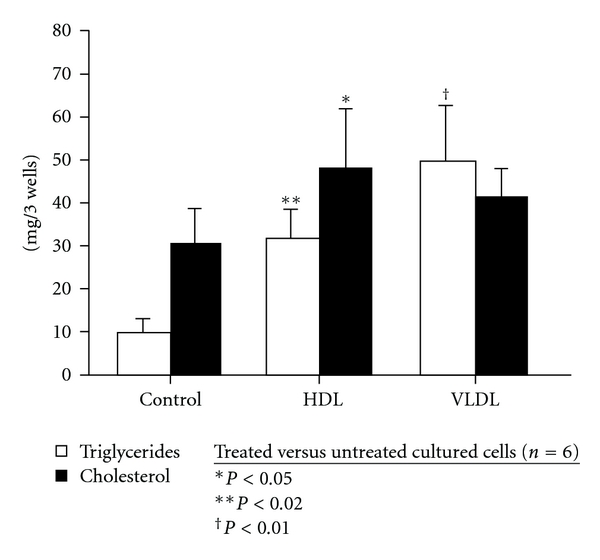
Triglyceride and cholesterol content of cultured sebocytes following treatment with HDL and VLDL. HDL induced significantly greater concentrations of both triglyceride and cholesterol, whereas VLDL induced a high level of triglyceride accumulation, but had no significant effect on cholesterol accumulation. Triglyceride (*n* = 5) and cholesterol (*n* = 6) were assayed per 3 wells. Means +/− SEMs are shown.

Since lipoproteins deliver cholesterol and fatty acids to cells, we tested the effect of lipoproteins on sebocyte differentiation. HDL 100 *μ*g protein/mL stimulated significantly more sebocyte LFCs (89 ± 3% SEM; *P* < 0.001) than any PPAR agonist (Figure 1). VLDL 100 *μ*g protein/mL tended to induce even more LFCs (99 ± 1%). In addition, sebocytes exhibited distinct patterns of cholesterol and triglyceride accumulation following treatment with HDL and VLDL (Figure 2). HDL stimulated predominantly cholesterol accumulation, along with a lesser, but significant, triglyceride accumulation. VLDL stimulated primarily triglyceride accumulation. These patterns of lipid accumulation reflect the distinct core lipid composition of these two lipoproteins [23]: HDL 100 *μ*g protein/ml contains approximately 100 *μ*g lipid/mL, of which the core lipid constitutes about half and consists of approximately 78% cholesteryl ester and 22% triglyceride, while VLDL 100 *μ*g protein/mL contains approximately 1000 *μ*g lipid/mL, of which the core lipid constitutes about three-quarters and consists of approximately 18% cholesteryl ester and 82% triglycerides. Thus, HDL and VLDL appear to act in part by delivering substrate for lipogenesis. However, this is probably receptor mediated for reasons discussed below.

### 3.4. Evidence that the Apoprotein Component of HDL and VLDL Plays a Role in Sebocyte Differentiation

**Figure 3:**
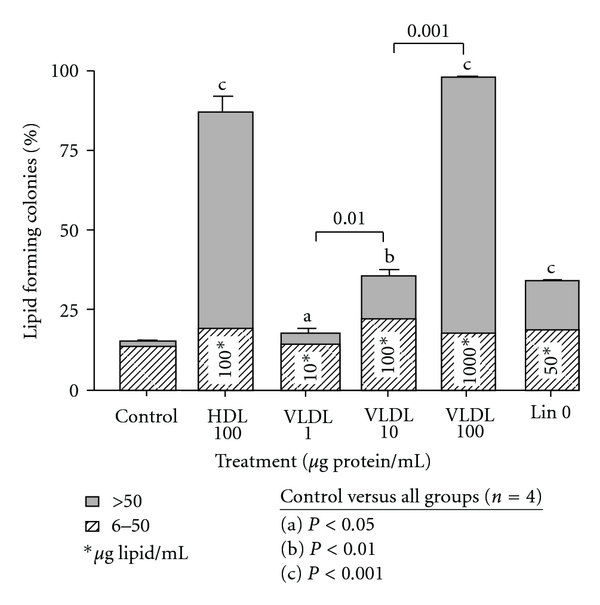
Comparison of protein versus lipid content of HDL, VLDL, and LIN on induction of sebocyte differentiation. VLDL was more potent than HDL in the induction of sebocyte differentiation in terms of protein content but less potent when comparing lipid content (*n* = 4). Means +/− SEMs are shown.

VLDL had a dose-response effect on sebocyte differentiation over the range 1–100 *μ*g protein/mL (Figure 3). VLDL was as effective in inducing LFCs as HDL at a comparable apoprotein dosage of 100 *μ*g protein/mL but was less effective at an equivalent lipid concentration (100 *μ*g lipid/mL). 

HDL was more effective than linoleic acid at a dose that had about 8% triglyceride content. Despite using submaximal doses as low as 25 *μ*g protein/mL (about 25 *μ*g lipid/mL), HDL was significantly more potent than linoleic acid, 200 *μ*g/ml (100 *μ*M; data not shown). VLDL was as effective in inducing LFCs as a dose of linoleic acid that delivers approximately half the amount of fatty acid (Figure 3). Notably, unlike linoleic acid, neither lipoprotein significantly stimulated LFCs in keratinocytes, suggesting a sebocyte-specific effect (data not shown). In addition, human LDL had no significant effect on sebocyte differentiation in our sebocyte culture system (data not shown). These considerations suggest that the apoprotein component of lipoproteins plays a key role in sebocyte differentiation and that the mechanism of lipoprotein action is not simply due to passive delivery of lipid substrate.

### 3.5. PPAR*δ* and PPAR*γ* Antagonists Attenuate Lipoprotein Effects on Sebocyte Differentiation

Both PPAR*γ* antagonists significantly inhibited LFC formation in response to HDL or VLDL (Figure 1). This suggests that not only do HDL and VLDL deliver lipid substrate to sebocytes, but also PPARs partially mediate the effects of these lipoproteins on induction of sebocyte differentiation. This conclusion is supported by the finding that HDL and VLDL effects were not discernibly augmented by PPAR agonists (data not shown), which is consistent with action via common pathways. The data are compatible with the concept that HDL and VLDL supply one or more critical signaling molecules that act downstream of PPAR*γ* action, perhaps through the same PPAR*γ*-independent pathways stimulated by PPAR*δ*.

### 3.6. Sebocytes Express a Unique Pattern of Lipogenic Genes

**Figure 4:**
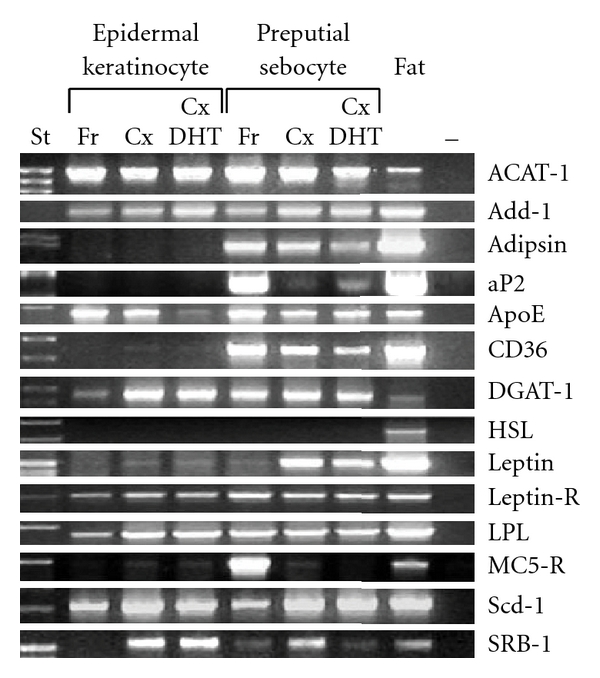
Comparison of the expression of lipogenic regulatory genes in freshly dispersed (Fr) and cultured (Cx) ± dihydrotestosterone (DHT) preputial sebocytes and epidermal cells, using RT-PCR. Total RNA was isolated from adult rat preputial sebocytes, epidermal cells and homogenized epididymal fat pad. Primers specific for the listed genes were used (see Table 1). Adipsin, aP2, CD36, and MC5-R were only detected in sebocytes, not in fresh or cultured epidermal cells. Leptin was detected in cultured sebocytes (less mature) to a greater extent than in freshly dispersed sebocytes (more mature) or epidermal cells. MC5-R was detected in freshly dispersed sebocytes to a greater extent than cultured sebocytes.

The role of PPARs and lipoproteins in sebocyte differentiation suggests that the mode of molecular genetic regulation of lipogenesis in sebocytes may be different in some respects from that in fat cells. Lipogenic gene expression was therefore explored by RT-PCR (Figure 4). Sebocytes were found to have a unique molecular fingerprint of lipogenic gene expression. Adipsin, adipocyte fatty acid binding protein (aP2), fatty acid translocase (CD-36), and melanocortin 5 receptor (MC5-R) were only detected in sebocytes, not in epidermal cells. Leptin was detected in cultured sebocytes (which are immature) to a greater extent than in freshly dispersed sebocytes (which are predominantly mature) or epidermal cells. The higher leptin gene expression in cultured than in freshly dispersed cells suggests that leptin expression may be upregulated early in differentiation then downregulated in late-differentiated sebocytes. In contrast, MC5-R was detected in freshly dispersed sebocytes but not in cultured sebocytes. MC5-R thus appears to be a marker of a later stage of differentiation than is achieved in culture since it is found only in freshly dispersed sebocytes. Other lipogenic genes including acyl-CoA:cholesterol acyltransferase type 1 (ACAT-1), adipocyte determination factor (Add-1), apolipoprotein E (ApoE), acyl-CoA:diacylglycerol acyltransferase type 1 (DGAT1), leptin receptor, lipoprotein lipase (LPL), stearoyl-CoA desaturase type 1 (Scd-1), and scavenger receptor type BI (SR-B1) were expressed in both sebaceous cells and epidermal cells at all stages of maturation. Androgen (dihydrotestosterone) had no clear effect on expression of any gene tested.

## 4. Discussion

**Figure 5:**
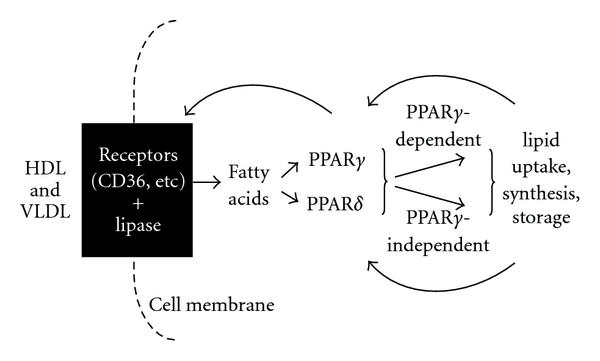
Feed-forward model of sebocyte differentiation. We postulate that lipoproteins target lipid substrates within skin to sebocytes through interactions with specific receptors. The substrates in turn stimulate sebocyte differentiation by acting through cell-specific PPAR*γ*-mediated lipogenic pathways.

Our studies show that the lipoproteins HDL and VLDL are the most effective inducers yet reported of rat sebocyte colony differentiation. Our results also indicate that PPAR*δ* plays a unique role in sebocyte differentiation. Notably, HDL, VLDL, and PPAR*δ* all boost lipogenesis in a seemingly PPAR*γ*-dependent manner. Our data are consistent with a model in which lipoproteins specifically target lipids within skin to sebocytes, where these lipids serve a dual role, acting both as substrate and to generate signaling molecules that induce differentiation via cell-specific PPAR-mediated lipogenic pathways (Figure 5).

Lipoproteins interact with cells by binding of their apoprotein to receptors, allowing for transport of their constituent lipids into cells where they provide lipids both as substrates for intracellular metabolism and as signaling ligands for PPARs. Thus, regardless of their intracellular function, their initial interaction involves association with cell surface receptors, which could discriminate among particular classes of lipoproteins. The potential importance of this receptor interaction is suggested by the selectivity of HDL and VLDL for sebocyte differentiation in comparison to linoleic acid, which also acts as both a lipid substrate and a PPAR agonist, yet indiscriminately induces lipid droplet formation in both sebocytes and keratinocytes [1]. Our data suggest that the effectiveness of the lipoproteins is due to a regulatory effect of the apoprotein moiety rather than the simple delivery of substrates. The importance of receptor interaction is further supported by the finding that VLDL is at least, if not more potent than HDL when matched for protein concentration, but less potent when presented at similar lipid concentrations. The lack of induction of sebocyte differentiation by human LDL militates against the possibility of lipoproteins passively transferring lipid to target cells. Paradoxically, this finding is compatible with a role for the LDLR in mediating lipoprotein action, since the rodent LDL receptor has a much lower affinity for human LDL than it has for rodent LDL [24].

The role of PPAR*δ* in sebocyte differentiation is unique. Not only is PPAR*δ* the predominant PPAR isoform in sebocytes [25], but the specific PPAR*δ* agonists GW742 and L-165041 also are as potent as the previously described PPAR*δ*, *α* agonist cPGI2 and more potent than 100-fold higher concentrations of linoleic acid. Furthermore, our data support the concept that PPAR*δ* stimulates lipogenesis via both PPAR*γ*-dependent and-independent pathways. We found that the modest effect of a low dose of the specific PPAR*δ* agonist GW742 could not be blocked by the specific PPAR*γ* antagonist GW5393 that blocked the more marked effect of high dose GW742 stimulation. This suggests that high-dose PPAR*δ* stimulation activates PPAR*γ* signaling pathways like those involved in linoleic acid action: linoleic acid, a direct PPAR*δ*, *α* agonist, functions as an indirect PPAR*γ* agonist in adipocytes [26] and in macrophages via its oxidized metabolites 9- and 13-HODE [7].

Although PPAR*δ* appears to be important in sebocyte differentiation, the specific PPAR*γ* agonist GW845 and HDL and VLDL are more effective inducers of sebocyte differentiation. PPAR*γ* signaling seems critical for the action of both lipoproteins and all other PPAR isoform agonists, since the specific PPAR*γ* competitive antagonist GW5393 inhibits the effects of all substantially. 

The pattern of PPAR effects in sebocytes differs from that in other model systems (Table 2). The PPAR*γ* agonist stimulation of sebocyte lipid metabolism resembles that in fat cell (adipocyte) differentiation but is opposite to the effects on macrophages, in which PPAR*γ* promotes lipid efflux rather than lipid storage [13, 27, 28]. On the other hand, PPAR*δ* agonist stimulation of sebocyte lipid formation is similar to that found in the macrophage, where PPAR*δ* agonist induces storage of lipids derived from oxLDL and serum [13], and contrasts to its action in adipocytes where it has equivocal effects on lipid storage and the overall net effect of promoting lipid catabolism [29]. 

Our studies also demonstrate that sebocytes have a characteristic molecular fingerprint: unlike keratinocytes, they expressed adipsin, aP2, CD36, and MC5-R, and immature sebocytes strongly expressed leptin, all of which play important roles in lipogenesis. The unique expression pattern of CD-36 makes it a candidate receptor to explain the specificity of HDL for promoting sebocyte lipogenesis in skin. CD-36 acts as a lipoprotein receptor and mediates the transport of long-chain fatty acids in mature adipocytes, macrophages, and other tissues following HDL binding [12, 30–32]. CD-36 expression has also recently been demonstrated on the surface of SZ95 human sebocytes where it is thought to play a role in the transport of free fatty acids [33]. The scavenger receptor SR-B1 acts similarly, but its skin expression pattern is not confined to sebocytes; thus it would not seem to be a candidate for mediating the sebocyte-specific action of lipoproteins. 

This molecular fingerprint further suggests that expression of a unique pattern of lipogenic genes underlies sebocyte differentiation. Adipsin is used by adipocytes to determine their rate of glycerol incorporation into fatty acids [34]. aP2 is an intracellular fatty acid transport protein that appears to play a role in intracytoplasmic fatty acid trafficking [35, 36] and is induced by PPARs [13]. MC5-R, an appetite suppressive hormone involved in the regulation of adipose stores, has been identified in preputial and human sebaceous glands [37, 38], where it mediates the augmentation of androgen action by melanocyte stimulating hormone-*α* [39, 40]. Disruption of the MC5-R gene in mice results in hypoplasia of sebaceous and preputial glands with decreased sebum production [41]. Leptin is a hormone secreted by adipocytes that is a key metabolic satiety signal in hypothalamic appetite control [42] and may downregulate fatty acid desaturation [43]. MC5-R and leptin seem to be expressed at distinctly different time points during sebocyte differentiation. MC5-R mRNA is expressed primarily in the highly differentiated sebocytes of freshly dispersed sebocytes, but little if at all in cultured (immature) sebocytes or epidermal cells. In contrast, we found leptin mRNA to be expressed in immature sebocytes, but it was consistently barely detectable in mature sebocytes and cultured and freshly dispersed epidermal cells. Remarkably, with the exception of MC5-R, these genes are not among the lipogenic genes previously identified as essential for sebogenesis, all of which are expressed in keratinocytes as well as sebocytes: ACAT-1 [44, 45], DGAT-1 [46], and Scd-1 [47, 48]. 

Interestingly, addition of androgen (dihydrotestosterone) to our culture system had no clear effect on expression of any gene tested. Whereas sebaceous gland growth has been shown to be dependent on androgen in vivo, androgens alone have not been shown to have a clear effect on sebocyte differentiation in vitro [2, 4, 49]. Androgens, however, have been shown to enhance the effect of PPAR agonists on sebocyte differentiation. Nevertheless, there is no convincing evidence that this suffices to bring about complete maturation of sebaceous cell differentiation in vitro. Consequently, the mechanism by which androgens bring about full sebaceous gland development is not yet fully understood.

## 5. Conclusions

On the basis of these studies, we propose a feed-forward mechanism of sebocyte differentiation that operates through PPAR*γ*-dependent and PPAR*γ*-independent pathways (Figure 5). HDL and VLDL are the most potent inducers of sebocyte differentiation and are both more effective than explicable by their lipid content, suggesting that their apoproteins moieties mediate their effects. In addition, HDL and VLDL are selective for the induction of sebocyte differentiation, as compared to keratinocyte differentiation. These observations suggest the involvement of specific lipoprotein receptors, such as CD-36, in activating signaling pathways in a hierarchy that is specific for sebocytes. In turn, accumulating lipids, such as linoleic acid itself, seem to act as both substrates and agonists of PPAR*δ* and PPAR*γ* to amplify multiple lipid metabolic pathways for net lipid uptake and storage including further PPAR*γ*-dependent stimulation of CD36 expression [7–9, 13]. 

There is still much unknown about sebocyte differentiation. Elucidation of the mechanism of sebocyte lipogenesis will require a further understanding of the coordinated function of the many requisite genes and gene products.

## Figures and Tables

**Table 1:** Primers for RT-PCR.

	Primer sequence	
Primer	Forward (5′ to 3′)	Reverse (5′-3′)
ACAT-1	5′-ATGGCTGCCCTGGCGGTTCTA-3′	5′-CTACAGCTTCTCAATCAGCAC-3′
Add-1	5′-ATGGATTGCACATTTGAAGAC-3′	5′-TGGTACTGTGGCCAGGATGGT-3′
Adipsin	5′-ATGCACAGCTCCGTGTACCTC-3′	5′-TCAGGCCGTCACGTTAACACT-3′
aP2	5′-TGGAAACTCGTCTCCAGTGAG-3′	5′-CAAATTTCAGTCCAGGGCCTC-3′
Apo-E	5′-ATGAAGGCTCTGTGGGCCCTG-3′	5′-TCATTGATTTCTCCAGGGCAC-3′
CD36	5′-GCAGCTGCACCACATATCTAC-3′	5′-GCTGGCTTGACCAGTATGTTG-3′
DGAT-1	5′-GCGGTTTCAGCAATTACCGTG-3′	5′-ACTGGGGCATCATAGTTGAGC-3′
HSL	5′GAAACCTAGGAGACCAATTTC-3′	5′-ACCTGCAAAGACGTTGGACAG-3′
Leptin	5′-ATGACATTTCACACACGCAGT-3′	5′-CTAGAGGAGTAGGAGAAACGG -3′
Leptin-R	5′-ATGACGTGTCAGAAATTCTATG-3′	5′-GGAAGCATTGGATCCAACACT-3′
LPL	5′-ATGGAGAGCAAAGCCCTGCTC-3′	5′-CAGAGACTTGTCATGGCATTT-3′
MC5-R	5′- ATGAACTCCTCGTCTCACCTG -3′	5′- TTAATACCTGCCAAGGAGCGT -3′
Scd-1	5′-ATGCCGGCCCACATGCTCCAA-3′	5′-TCAGCTACTCTTGTGGCTCCC-3′
SR-B1	5′-ATCATGATTCTCATGGTGCCC-3′	5′-TGGCAGCTGGTGACATCAGAG-3′

acyl-CoA:cholesterol acyltransferase type 1 (ACAT-1), adipocyte determination factor (Add-1), adipocyte fatty acid binding protein (aP2), apolipoprotein E (ApoE), fatty acid translocase (CD-36), acyl-CoA:diacylglycerol acyltransferase type 1 (DGAT1), hormone sensitive lipase (HSL), leptin receptor (Leptin-R), lipoprotein lipase (LPL), melanocortin 5 receptor (MC5-R), stearoyl-CoA desaturase type 1 (Scd-1), and scavenger receptor type BI (SR-B1).

**Table 2:** Comparison of effects of PPAR agonists and lipoproteins on lipid storage in adipocytes, macrophages, and sebocytes.

Substrates	Adipocyte	Macrophage	Sebocyte
PPAR*γ*	positive	negative	positive
PPAR*δ*	positive/negative	positive	positive
LDL	?	positive (oxLDL)	no effect
HDL	?	?	positive
VLDL	positive	?	positive

oxLDL: oxidized low-density lipoprotein.

## Abbreviations

ACAT-1: acyl-CoA: cholesterol acyltransferase type 1
Add-1: adipocyte differentiation and determination factor-1
aP2: adipocyte fatty acid binding protein
ApoE: apolipoprotein E
DGAT1: acyl-CoA: diacylglycerol acyltransferase type 1
cPGI2: carbaprostacyclin
CD-36: fatty acid translocase
HDL: high density lipoprotein
HSL: hormone sensitive lipase
Leptin-R: leptin receptor
LFCs: lipid-droplet forming colonies
LPL: lipoprotein lipase
LDL: low-density lipoprotein
MC5-R: melanocortin 5 receptor
PPAR: peroxisome proliferator- activated receptor
Scd-1: stearoyl-CoA desaturase type 1
SR-B1: scavenger receptor type BI
Tro: troglitazone
VLDL: and very low-density lipoprotein.
